# Integrative Transcriptomic and Interactome Analyses Prioritize Candidate Genes for Alzheimer’s Disease

**DOI:** 10.1007/s12035-026-06258-0

**Published:** 2026-10-10

**Authors:** Giovanni Monteiro Maglio, Nicolly Clemente de Melo, Lucas Miguel de Carvalho

**Affiliations:** São Francisco University, Av. São Francisco de Assis, 218, Bragança Paulista, SP 12916-900 Brazil

**Keywords:** Alzheimer’s disease, Bioinformatics, Neuroinflammation, Brain aging

## Abstract

Alzheimer’s disease (AD) is a heterogeneous neurodegenerative disorder characterized by complex molecular alterations and diverse cognitive, behavioral, and neuropsychiatric manifestations. Although transcriptomic studies have identified several pathways associated with AD, the integration of molecular signatures with disease-related phenotype annotations and pathological aging remains incompletely understood. Therefore, this study aimed to integrate transcriptomic profiling, protein–protein interaction (PPI) network analysis, pathological aging assessment, and curated clinical phenotype annotations to prioritize candidate molecular signatures associated with AD. Differential expression analyses and network-based approaches were applied to publicly available transcriptomic datasets derived from distinct brain regions. Candidate genes and pathways were prioritized through interactome reconstruction, functional enrichment analyses, cluster-based analyses, and independent validation in an external dataset. In addition, transcriptomic signatures were integrated with curated clinical phenotype annotations using a knowledge-based gene overlap strategy. The analysis identified molecular signatures associated with neuroinflammatory signaling, synaptic dysfunction, endocytosis, and pathological aging. Pathways related to MAPK, PI3K/Akt, NF-κB, and TGF-β1 signaling emerged as candidate dysregulated pathways in AD. Comparative analyses of physiological aging and the AD aging comparison enabled the prioritization of candidate genes potentially more closely associated with the AD condition than with aging alone, including *FOS* and *TAB2*. Independent validation reinforced the reproducibility of several prioritized genes, including *GFAP*,* CXCR4*,* CCL2*,* HSPA1A*, *FCGR2A*, *TLR2*, *CD44*,* TLR4*,* FOS*, *TAB2*,* CDC42*, and *SH3GL2*. Integration with curated clinical phenotype annotations suggested partially overlapping molecular signatures across neuropsychiatric and cognitive phenotype annotations in AD. Together, these findings demonstrate the utility of integrative bioinformatics approaches for prioritizing candidate genes, pathways, and molecular signatures associated with AD. This systems-level framework may contribute to future mechanistic studies aimed at understanding the molecular heterogeneity of AD and its associated phenotypic complexity.

**Supplementary Information:**

The online version contains supplementary material available at https://doi.org/10.1007/s12035-026-06258-0.

## Introduction

Alzheimer’s disease (AD) is the leading cause of dementia, characterized by progressive cognitive decline, which usually begins with the loss of the ability to form new memories and eventually compromises all intellectual functions. The progression of the disease leads to total dependence for basic activities of daily living and is associated with increased mortality [1, 2]. Various clinical manifestations have been related to AD, which compromise patients’ quality of life. Among the main symptoms are memory loss, confusion, cognitive decline, and progressive dementia [3–5]. Behavioral changes are also common, including aggressiveness, apathy, distrust, irritability, anxiety, restlessness, and agitation [6–10].

The AD progression can lead to more severe manifestations, such as seizures, psychosis, depression, euphoria, disinhibition, and sleep disorders [7, 11, 12]. Other frequently observed symptoms include sundowning syndrome, wandering, stereotyped movements, personality changes, and difficulties with eating behaviors [13–17]. Furthermore, functional incapacity becomes increasingly evident, requiring constant care adapted to the progression of the disease [7]. The prevalence of the disease increases exponentially with age, becoming significantly higher after 65 years, with an increase of almost 15 times between the ages of 60 and 85 [2].

Genetic factors play a relevant role in the risk and progression of AD. Although the familial early-onset form is rare, the sporadic late-onset form represents the vast majority of cases, and genetic susceptibility factors are recognized as important contributors [18]. This genetic background, in combination with environmental and lifestyle factors, contributes to the clinical and biological heterogeneity observed in the disease [19].

In recent years, bioinformatics and systems biology have emerged as fundamental approaches for investigating complex molecular networks in neurodegenerative diseases. These fields combine techniques for discovering, organizing, and analyzing large volumes of biological data [20], allowing the in silico modeling of molecular interaction networks and gene regulatory mechanisms [21, 22]. Modern computational tools enable the integration of transcriptomics, proteomics, and clinical data to suggest molecular signatures and regulatory patterns that can elucidate the dynamics and functionality of biological systems involved in neurodegeneration [20, 23–25].

Among bioinformatics approaches, protein–protein interaction (PPI) analysis has stood out as a promising methodology for studying AD. Several recent works have used PPI networks to prioritize central proteins associated with disease pathogenesis, as well as potential targets for future mechanistic and therapeutic investigation [26–28]. Likewise, the integration of PPI analysis with other computational approaches, such as functional enrichment and drug interaction prediction, has allowed the proposal of new compounds with therapeutic potential for the disease [27, 28].

Although numerous transcriptomic and network-based studies have investigated molecular alterations associated with AD, most approaches focus primarily on differential gene expression or pathway enrichment without integrating these findings with curated clinical phenotype annotations associated with disease heterogeneity [29–31]. In particular, neuropsychiatric and cognitive manifestations such as psychosis, depression, anxiety, aggression, apathy, and memory impairment are highly prevalent in AD and substantially impact disease progression and patient quality of life, yet their underlying molecular signatures remain incompletely understood [32]. Furthermore, few studies have combined transcriptomic profiling, protein–protein interaction network analysis, pathological aging comparisons, independent validation, and phenotype annotation mapping within a unified analytical framework [33, 34]. Therefore, integrative approaches capable of mapping transcriptomic alterations onto curated clinical phenotype annotations may contribute to link systems-level understanding of the biological complexity and phenotypic heterogeneity associated with AD.

Based on this rationale, the present study aimed to investigate whether the integration of transcriptomic analyses, protein–protein interaction networks, and curated clinical phenotype annotations could prioritize biologically relevant molecular signatures associated with AD heterogeneity. We hypothesized that integrating these analytical layers within a systems-level framework would allow the identification of candidate molecular pathways potentially relevant to core neurodegenerative mechanisms and to distinct neuropsychiatric and cognitive phenotype annotations associated with AD.

## Materials and Methods

**Fig. 1:**
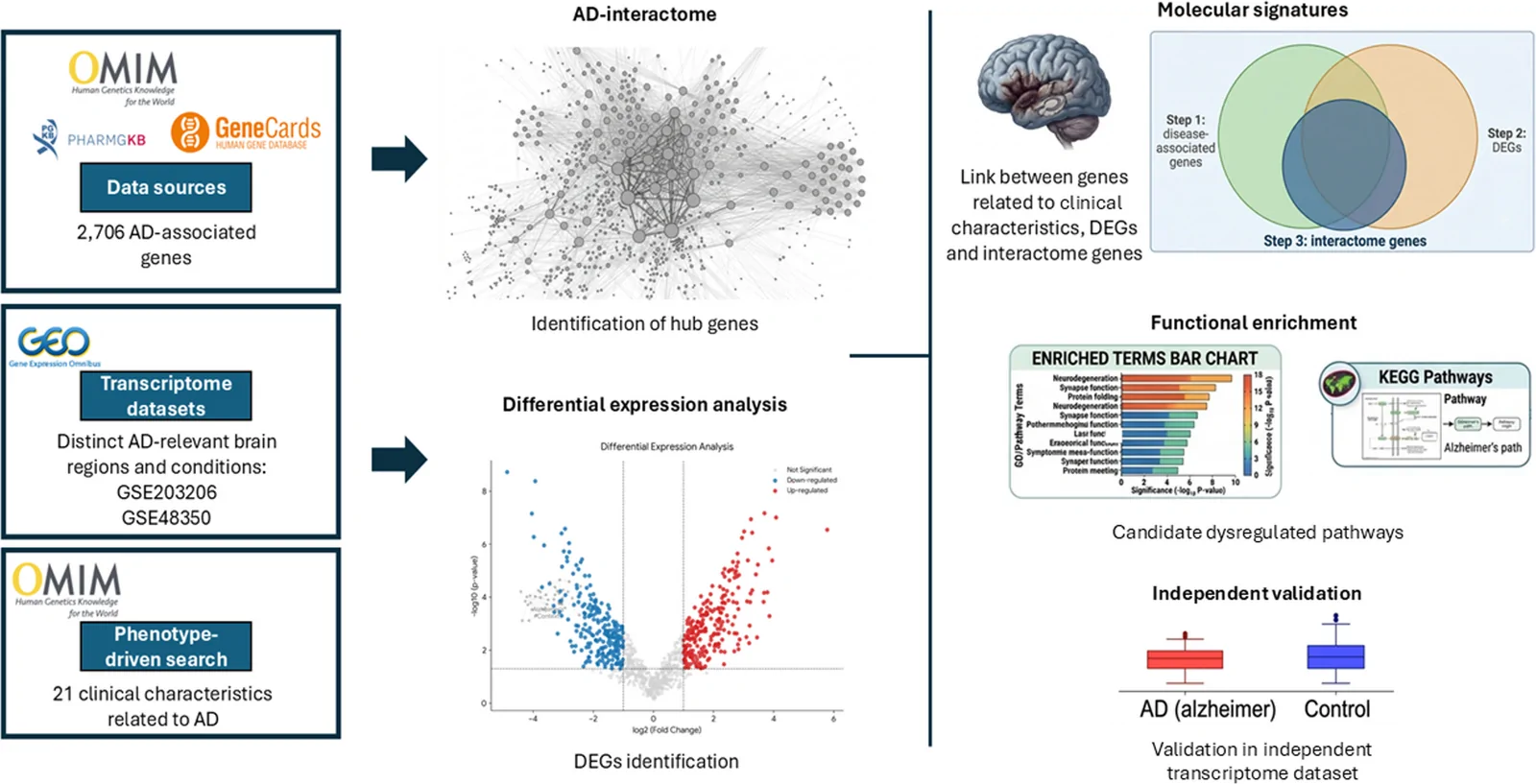
Overview of the integrative analytical framework used in this study. Publicly available AD-associated genes, transcriptomic datasets, and curated clinical phenotype annotations were integrated through a multi-step bioinformatics workflow. AD-associated genes obtained from GeneCards, OMIM, and PharmGKB were used to construct an AD protein–protein interaction (PPI) network and identify hub genes and network modules. Transcriptomic datasets derived from distinct AD-relevant brain regions and conditions were independently analyzed to identify differentially expressed genes (DEGs) associated with AD subtypes, physiological aging, and pathological aging. Molecular signatures were prioritized by integrating disease-associated genes, DEGs, and interactome genes, together with clinical annotations derived from phenotype-driven searches. Functional enrichment analyses were subsequently performed to identify candidate dysregulated pathways and biological processes. Finally, prioritized molecular signatures were evaluated in an independent transcriptomic dataset to assess the reproducibility of their expression patterns

We implemented an integrative bioinformatics pipeline combining transcriptomic datasets and network analysis to elucidate molecular features of Alzheimer’s disease (AD) and their overlap with curated clinical phenotype annotations (Fig. 1). Initially, a comprehensive gene set associated with AD was compiled from public databases (GeneCards, OMIM, PharmGKB) to construct a protein–protein interaction (PPI) network, which was topologically analyzed and clustered to identify hub genes and key modules. Concurrently, transcriptomic data from the Gene Expression Omnibus (GEO) repositories (GSE203206 and GSE48350) were processed to identify differentially expressed genes (DEGs) across distinct clinical and aging groups. The intersection of network topology and differential expression prioritized robust candidate genes, which were then independently validated using the GSE122063 dataset (external dataset). Subsequently, functional enrichment analysis (GO/KEGG) revealed dysregulated pathways, and a systematic correlation with a curated set of 21 clinical AD phenotypes was performed to evaluate the intersection between molecular signatures to curated clinical phenotype annotations derived from literature and database resources. All analyses were conducted using reproducible methodologies, with results detailed in the Supplementary Material.

### Search for Alzheimer’s Targets and Associated Clinical Characteristics

Genes associated with AD were collected from three databases: the top 2000 ranked genes from GeneCards, which provides comprehensive information on annotated human genes; OMIM, a detailed compendium of genes and genetic phenotypes; and PharmGKB, a database focused on pharmacogenomics. The selection was made considering only genes explicitly related to AD, according to the annotation in the title or description of the databases. A total of 930 genes were obtained from OMIM, 2000 genes from GeneCards, and eight genes from PharmGKB. After removing duplicated genes across the three sources, the datasets were combined into a non-redundant set of 2706 unique AD-associated genes. The overlap among the databases was also assessed, with 224 genes shared between OMIM and GeneCards, eight genes shared between OMIM and PharmGKB, and eight genes shared between GeneCards and PharmGKB; the eight genes shared in each pairwise comparison were also common to all three databases.

Because GeneCards retrieves a large number of genes with varying levels of evidence for disease association, only the top 2000 genes ranked by GeneCards relevance score were selected for downstream analyses [35]. This threshold was defined to balance comprehensiveness and specificity, prioritizing genes with stronger evidence of association with AD while reducing network complexity and minimizing the inclusion of low-confidence candidates. The resulting gene set was subsequently integrated with OMIM and PharmGKB data to increase coverage and robustness.

### Construction and Analysis of the Protein–Protein Interaction (PPI) Network

For the analysis of the biological interactions of these genes, the STRING v12.0 database was used, which integrates known and predicted associations between proteins, including physical and functional interactions [36]. The PPI network of potential targets was constructed using the following conditions: the selected species was “Homo sapiens” and the confidence score was “highest confidence ≥ 0.9.” Subsequently, the PPI network was analyzed using Cytoscape v3.8.0 software [37] to obtain topological parameters of the network nodes. The CytoHubba plugin was employed to identify hub genes based on the degree value, which indicates the number of direct interactions a node has and is commonly used to assess the node’s importance in networks. A gene was considered a “hub gene” if it was among the top 10 genes with the highest degree value. The MCODE plugin was used for PPI network module clustering, where submodules with a score greater than 6.0 were selected.

### Transcriptomic Data Analysis

For the analysis of differential gene expression in AD, publicly available transcriptomic datasets generated using RNA-seq and microarray platforms were obtained from the Gene Expression Omnibus (GEO). The selected datasets are:

a GSE48350: This dataset contains microarray information from four distinct brain regions: hippocampus, entorhinal cortex, superior frontal cortex, and postcentral gyrus. The study compared gene expression of healthy individuals (aged 20 to 99 years) and AD patients. For this study, only the hippocampus samples were used, as this is a crucial region and one of the most affected by AD. The samples were further divided into three groups based on age: young, elderly without AD, and elderly with AD, allowing a more detailed analysis of gene expression changes related to aging and pathology.

To descriptively distinguish transcriptional alterations observed during physiological aging from those preferentially detected in the AD aging comparison, genes and pathways identified in both the healthy aging comparison (elderly without AD versus young individuals) and the AD aging comparison (elderly with AD versus young individuals) were excluded from the latter analysis. The remaining molecular alterations were retained as candidate features preferentially observed in the AD aging comparison. This overlap-based approach was used as a descriptive comparison of group-specific DEG and pathway lists and was not intended to formally test an age x disease interaction.

b GSE203206: This study focused on the transcriptomic analysis of the primary visual cortex of patients with early-onset AD (EOAD) and late-onset AD (LOAD), comparing them to healthy individuals. Forty-eight samples were selected, comprising eight healthy controls and 40 AD patient samples (19 EOAD and 21 LOAD). The study investigated the molecular variability of the disease and indicated that transcriptomic profiles may not be directly related to the age of pathology onset.

Differential expression analyses were performed to identify differentially expressed genes across the different AD-related datasets, focusing on molecular signatures associated with the previously defined curated clinical phenotype annotations. Depending on the dataset, the analyses enabled comparisons between distinct disease stages, age groups, and pathological conditions associated with AD. The GEO2R web platform, which is based on the limma R package for transcriptomic data analysis, was used for all differential expression analyses, and to obtain the the normalized expression matrices. Log2 transformation was automatically detected and applied using the GEO2R default settings. Statistical comparisons between biological groups were performed using moderated *t*-tests implemented in limma, and *p* values were adjusted for multiple testing using the Benjamini–Hochberg (BH) false discovery rate (FDR) correction. Genes were considered differentially expressed when they met the thresholds of adjusted* p* value (FDR) < 0.05 and |log2 fold change|≥ 1. Voom precision weights were applied according to the GEO2R default configuration.

Each GEO dataset was analyzed independently using its corresponding normalized expression matrix and platform annotation. Differential expression analyses were performed according to the biological groups defined in the original studies, avoiding direct integration of datasets generated on different experimental platforms. This approach was adopted to minimize technical biases associated with cross-platform variability and to preserve the biological characteristics of each dataset. Additional variables such as age, biological sex, post-mortem interval (PMI), disease severity, and batch effects were not incorporated into a unified statistical model because these parameters were not consistently available across all analyzed datasets. Therefore, the analyses focused on the primary biological contrasts established in each study (Table S1 in the Supplementary Material).

### Independent Validation of Candidate Genes

To assess the reproducibility and directional consistency of selected candidate genes, an independent validation was performed using the GSE122063 dataset (58,717 probes). This dataset consists of temporal lobe tissue samples from 28 individuals with AD and 22 healthy controls, making it suitable for direct comparison with the initial results. The GSE122063 dataset was processed using GEOquery v2.74.0 in R v4.4.2 based on the normalized expression matrix available in GEO. The normality of gene expression values was assessed independently for the AD and control groups using the Shapiro–Wilk test (*p* value ≥ 0.05). Genes meeting this assumption were compared between groups using a two-sided Welch’s *t*-test, which does not assume equal variances. When the normality assumption was not satisfied for either group, differences in gene expression were evaluated using the two-sided Wilcoxon rank-sum (Mann–Whitney *U*) test with continuity correction. For multiple hypothesis testing across all evaluated genes, *p* values were adjusted using the BH FDR procedure.

### Clinical Characteristics Related to Alzheimer’s

The curated clinical phenotype annotations associated with AD were identified from the scientific literature, considering widely described cognitive, behavioral, and motor symptoms. Furthermore, a search was conducted in the OMIM database to survey proteins related to these characteristics, allowing the analysis of their relevance in the molecular context of the disease.

The main clinical characteristics analyzed in this study include aggressiveness, agitation, anxiety, apathy, confusion, dementia, depression, disinhibition, difficulties with eating behaviors, distrust, euphoria, irritability, memory loss, personality changes, psychosis, restlessness, seizures, sleep disorders, stereotyped movements, sundowning syndrome, and wandering [3–12].

Phenotype-associated gene sets were initially evaluated through gene overlap analysis, in which genes shared between the AD interactome and phenotype-associated gene sets were considered candidate molecular intersections between the AD-associated gene set and specific phenotype annotations. To statistically assess these overlaps, one-sided Fisher’s exact tests were performed using all genes comprising the AD interactome as the background gene set. For each of the 21 clinical phenotype categories, phenotype-associated genes were restricted to those present in the AD interactome and compared with the differentially expressed genes identified in each of the four transcriptomic contrasts, likewise restricted to the same background gene set. The analysis considered combined up- and downregulated DEGs for each transcriptomic contrast. For each comparison, observed and expected overlap, fold enrichment, odds ratio, and Fisher’s exact test *p* value were calculated. Jaccard similarity was additionally calculated as the size of the intersection between the phenotype-associated DEG set and the DEG set divided by the size of their union. *p *values were adjusted for multiple comparisons using the Benjamini–Hochberg procedure. A total of 84 tests (21 phenotypes × 4 contrasts) were performed. Statistical significance was defined as BH-FDR < 0.05 (Table S2 in the Supplementary Material).

### Functional Enrichment

All potential therapeutic targets were subjected to Gene Ontology (GO) [38] and Kyoto Encyclopedia of Genes and Genomes (KEGG) [39] pathway enrichment analysis using the DAVID [40] database to identify related pathways and GO terms (Biological Process (BP)). A cutoff of FDR < 0.05 was considered for a process/pathway to be deemed significant.

For cluster-level analyses, functional enrichment was performed independently for the gene sets contained within each selected cluster. Therefore, the biological pathways reported for each cluster emerged directly from the enrichment results of the corresponding cluster-specific gene sets and were not predefined or manually selected.

## Results and Discussion

### Construction and Characterization of the Alzheimer’s Disease Interactome

**Fig. 2:**
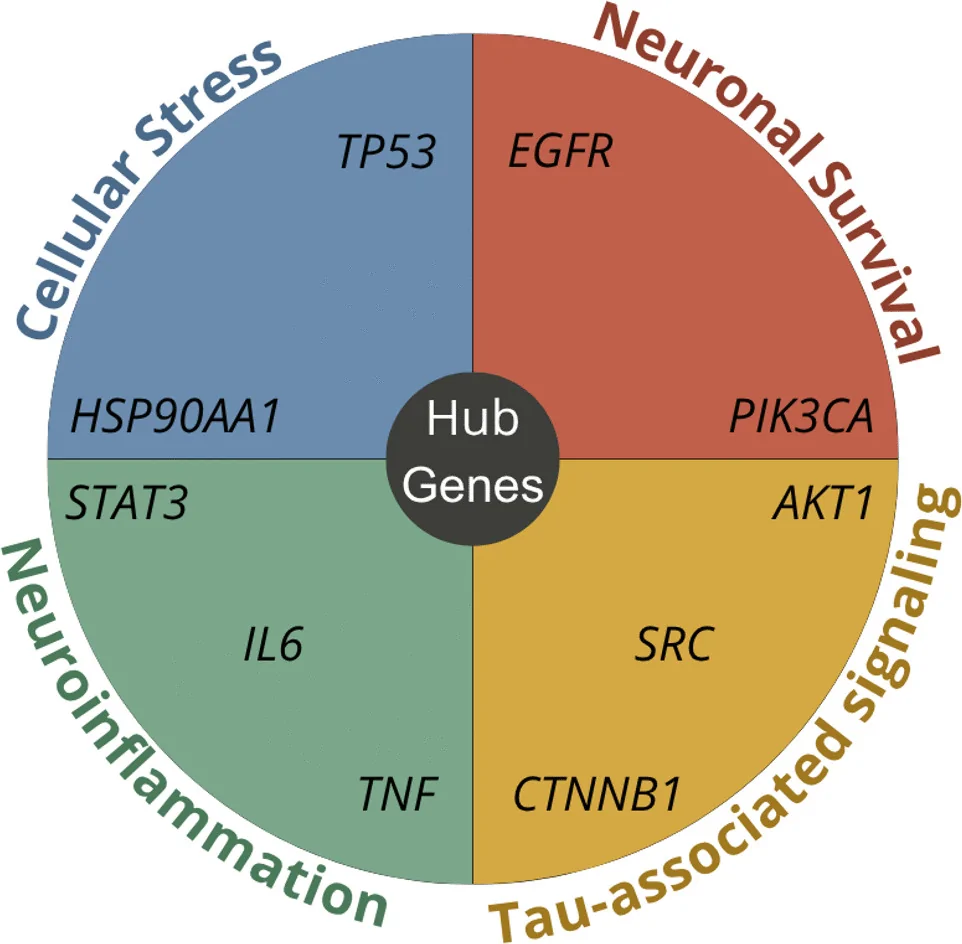
Functional categorization of prioritized hub genes identified in the AD interactome. Summarized are major biological processes associated with the principal hub genes identified through protein–protein interaction network analysis, including neuroinflammation, tau-related signaling, neuronal survival, and cellular stress. Hub genes were grouped according to their predominant biological roles reported in the literature and their relevance to AD-associated molecular mechanisms

After a search for AD-related genes, 2706 genes were found. Subsequently, using Cytoscape, a general disease interactome protein–protein interactome network was created using the STRING, resulting in a network containing 1751 proteins (nodes) and a total of 8974 interactions (edges). Next, using the CytoHubba tool, we identified the top 10 hub genes in the AD network (Table S3 in the Supplementary Material), which are *STAT3*, *SRC*, *AKT1*, *EGFR*, *TP53*, *CTNNB1*, *HSP90AA1*, *PIK3CA*, *TNF*, and *IL6*. The principal hub genes identified in the Alzheimer’s disease (AD) interactome and their associated biological functions are summarized in Fig. 2, revealing their involvement in four key pathological processes in AD. After this analysis, a search for submodules within the network was performed; these are highly interconnected regions that may represent significant importance in the disease pathology, where 11 submodules were found within the network (Table S4 in the Supplementary Material).

It is important to note that hub genes identified through protein–protein interaction network analyses should be interpreted cautiously. PPI databases are influenced by annotation and literature biases, and network centrality alone should not be interpreted as evidence of AD specificity genes. Instead, the identified hub genes should be viewed as prioritized candidates whose biological relevance requires interpretation in conjunction with previous experimental and clinical evidence.

The identification of highly connected genes such as *STAT3*, *TNF*, *IL6*, *AKT1*, *EGFR*, *TP53*, and *SRC* is consistent with their well-established roles in neuroinflammatory signaling, neuronal survival, amyloid processing, tau pathology, and neurodegeneration [41–51]. Given the known annotation and literature biases associated with PPI databases, these canonical hubs were considered primarily as biologically coherent components of the AD interactome rather than as novel AD-specific findings. Their identification therefore provides biological context for the disease-associated network but should not be interpreted as evidence of AD specificity based solely on network topology.

### Differential Transcriptomic Signatures in Early- and Late-Onset Alzheimer’s Disease

This analysis aimed to identify transcriptomic differences associated with early- and late-onset forms of AD and to prioritize molecular signatures potentially associated with disease heterogeneity.

To investigate whether early- and late-onset forms of AD exhibit distinct transcriptomic alterations associated with disease heterogeneity, differential expression analyses were performed using the GSE203206 dataset, which encompassed three different patient groups (see “Material and Methods”), and we performed two comparisons: EOAD vs. control patients and LOAD vs. control patients. Of these comparisons, only the EOAD vs. control showed differential genes, where 1014 upregulated, representing genes overexpressed in EOAD patients, of which 123 are present in the general AD interactome. Meanwhile, 266 downregulated were found, representing genes more expressed in healthy patients, of which 24 are in the general disease interactome (Table S5 in the Supplementary Material).

**Fig. 3:**
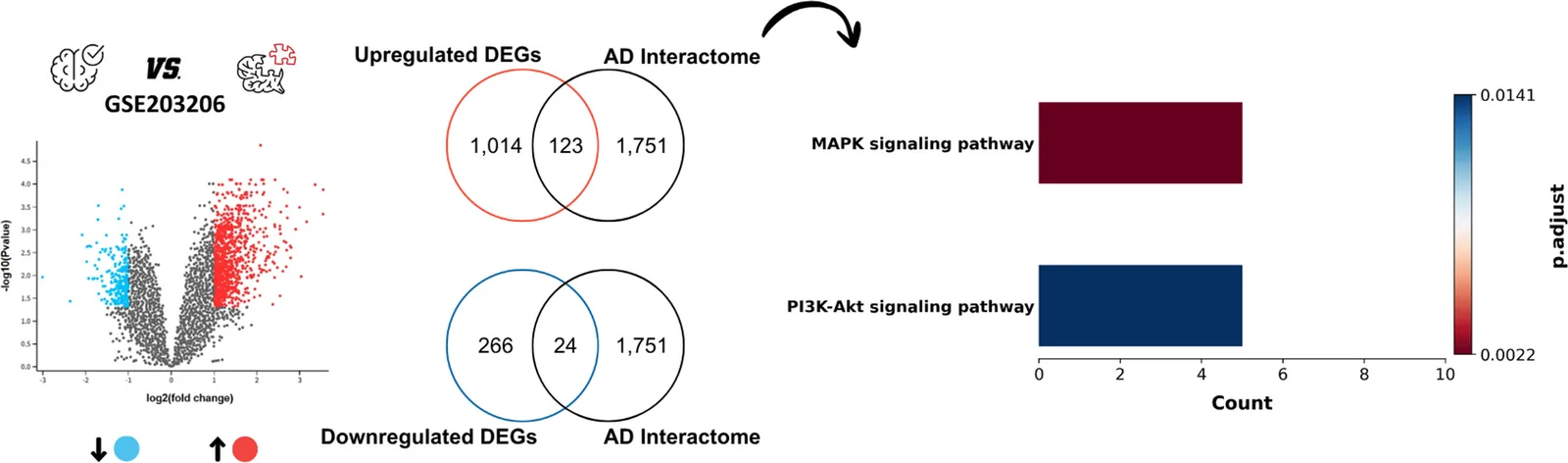
Overview of differential expression and functional enrichment analyses in the EOAD versus control comparison. Summarized are the transcriptomic workflow and principal findings identified in the comparison between early-onset AD (EOAD) patients and healthy controls from dataset GSE203206. The volcano plot illustrates the distribution of upregulated and downregulated differentially expressed genes (DEGs). Venn diagrams represent the overlap between DEGs and genes included in the AD interactome. Functional enrichment analysis of prioritized upregulated genes identified significant enrichment of MAPK and PI3K-Akt signaling pathways

An overview of the differential expression analysis, overlap with the AD interactome, and functional enrichment results identified in the EOAD versus control comparison is presented in Fig. 3.

The absence of significant DEGs in the LOAD vs. control comparison should be interpreted cautiously, and not be interpreted as evidence of a lack of molecular alterations, but rather as an indication that detectable transcriptomic differences were not identified under the analytical criteria applied in the present study. Although EOAD and LOAD samples were derived from the same dataset and analyzed using the same statistical framework, several factors may contribute to differences in the detected transcriptional signal, including biological heterogeneity within the LOAD group, variability in disease progression, and potential differences in the molecular processes associated with early- and late-onset forms of AD. Functional enrichment analysis of the upregulated (Table S6 in the Supplementary Material) genes revealed significant enrichment of the MAPK pathway. MAPK signaling has been associated with neurodegeneration, including effects on proinflammatory cytokine production by microglia and tau phosphorylation [52–54]. In the present analysis, the enrichment of MAPK-related genes is consistent with the previously reported involvement of this pathway in AD-related molecular processes, but does not directly establish pathway activation or its downstream functional consequences.

Among the upregulated genes associated with the MAPK pathway, *TGFBR2* and *HGF* overlapped with curated clinical phenotype annotations related to dementia, whereas *FLT4*, *NGFR*, and *HSPA1A* overlapped with annotations related to depression and anxiety (Table S7 in the Supplementary Material). These knowledge-based overlaps suggest that MAPK-related molecular alterations may be relevant to biological processes represented across cognitive and neuropsychiatric phenotype annotations. However, they should not be interpreted as evidence of direct gene–symptom relationships and require further experimental and clinical investigation [55, 56].

Another significantly enriched pathway was PI3K/Akt signaling, which has been widely associated with neuronal metabolism, cell survival, and differentiation [57]. The present enrichment results are consistent with evidence implicating alterations in PI3K/Akt signaling in AD pathophysiology [51]. Previous experimental studies have shown that PI3K/Akt signaling can regulate GSK-3β activity and thereby influence tau phosphorylation [46, 58]. However, GSK-3β activity, tau phosphorylation, and their downstream functional consequences were not directly assessed in the present study. Therefore, the enrichment of PI3K/Akt-related genes should be interpreted as supportive evidence of altered signaling associated with AD rather than as direct evidence of increased GSK-3β activity or tau pathology.

Among the upregulated genes contributing to the enrichment of the PI3K/Akt signaling pathway, *FLT4*, *NGFR*, *NOS3*, *HGF*, and *COL1A1*, which were upregulated in the EOAD dataset and present in the AD interactome, overlapped with distinct curated clinical phenotype annotations. *FLT4* and *NGFR* overlapped with annotations related to depression and anxiety, suggesting that modulation of the PI3K/Akt pathway may be related to molecular processes involved in neuronal circuits related to mood and stress response [59, 60]. The *NOS3* gene, in turn, overlapped with annotations related to memory loss, aggressiveness, dementia, anxiety, and psychosis [61, 62]. *HGF* overlapped with dementia-related annotations and has also been previously investigated in experimental AD models in the context of PI3K/Akt signaling [63–65]. Finally, *COL1A1* overlapped with annotations related to restlessness, a symptom frequently observed in patients with neurodegenerative disorders [66]. These relationships represent candidate knowledge-based associations rather than direct molecular explanations for the corresponding clinical manifestations.

The present enrichment analysis identified both PI3K/Akt and MAPK signaling among the significantly enriched pathways in the EOAD comparison. The concurrent identification of these pathways may indicate partially overlapping molecular processes associated with the transcriptomic alterations observed in EOAD. The involvement of PI3K/Akt and MAPK signaling in AD-related processes, including neuroinflammation, synaptic dysfunction, and neuronal survival, has also been documented [67]. Thus, the present findings are consistent with the established involvement of these pathways in AD pathophysiology but do not establish pathway activation, causal relationships, or downstream functional consequences. These pathways may therefore represent candidate molecular processes for further mechanistic investigation.

### Identification of Transcriptomic Signatures Associated with Physiological and Pathological Aging

This analysis aimed to distinguish transcriptomic alterations potentially associated with physiological aging from those preferentially associated with pathological aging in AD. Subsequently, the GSE48350 dataset was analyzed, which aimed to compare three groups of individuals: elderly individuals with AD (19 samples), elderly individuals without AD (25 samples), and young individuals (10 samples). Three pairwise comparisons were performed to characterize disease- and aging-associated transcriptional differences. The numbers of upregulated and downregulated DEGs, together with their overlap with the AD interactome, are summarized in Table 1, while the complete gene lists are provided in Table S8 of the Supplementary Material. These comparisons were subsequently used to distinguish molecular signatures associated with physiological aging from those preferentially observed in pathological aging.

**Table 1:** Summary of differential expression results obtained from the GSE48350 dataset

Group comparison	Biological context	Upregulated DEGs, *n* (AD interactome overlap)	Downregulated DEGs, n (AD interactome overlap)
Elderly with AD vs. elderly without AD	AD-related transcriptional differences	5 (1)	39 (7)
Elderly without AD vs. young individuals	Physiological aging	231 (57)	30 (3)
Elderly with AD vs. young individuals	Pathological aging	190 (37)	8 (1)

Upregulation and downregulation are reported relative to the first group indicated in each comparison. Values in parentheses indicate the number of DEGs overlapping with the AD interactome. Complete gene lists are provided in Table S8 of the Supplementary Material

Because the analyzed datasets were organized into predefined categorical groups, aging was investigated through group-based transcriptomic comparisons rather than continuous age modeling. Therefore, the identified molecular signatures should be interpreted within the context of physiological versus pathological aging contrasts rather than as linear age-associated transcriptional trajectories.

Differential expression analysis between healthy elderly and elderly with AD revealed three downregulated genes of the seven genes from the comparison that were in the interactome: *CDC42*, *AMPH*, and *SH3GL2*. These genes showed higher expression in healthy elderly brains, indicating that their higher expression in healthy elderly individuals may be consistent with previously described roles in neuronal homeostasis during aging [68–70]. Interestingly, although functional enrichment analysis of the complete set of downregulated genes did not identify any statistically significant pathway enrichment, manual inspection revealed that *CDC42*, *AMPH*, and *SH3GL2* are functionally related to endocytic processes. *CDC42* regulates cytoskeletal dynamics and vesicular transport [68], *AMPH* participates in synaptic vesicle endocytosis [69], and *SH3GL2* acts in clathrin-mediated endocytosis [70–73].

Among these genes, *CDC42* and *SH3GL2* emerged as particularly relevant candidate signatures associated with endocytic processes. The reduced expression of these genes in AD compared with healthy elderly individuals is consistent with their previously described roles in neuronal homeostasis and synaptic processes. Because no statistically significant pathway enrichment was identified, these findings should be interpreted as exploratory and hypothesis-generating rather than as evidence of a validated disease mechanism.

Endocytic dysfunction is recognized as a critical event in AD, contributing to the accumulation of β-amyloid and tau protein, impairing synaptic communication, and compromising neuronal survival [74]. The endocytosis pathway is essential for the internalization and recycling of membrane receptors and molecules, and its consistent deactivation in affected brains suggests impairment of synaptic homeostasis mechanisms and protein degradation [71–73]. Endocytic dysfunction has previously been associated with AD and may contribute to β-amyloid accumulation, tau pathology, synaptic dysfunction, and neuronal vulnerability [74–77]. Therefore, the coordinated downregulation of *CDC42*, *AMPH*, and *SH3GL2* observed in the present study may represent an exploratory indication of altered endocytic processes in AD. However, because no significantly enriched pathway was identified, this observation should be interpreted as hypothesis-generating rather than as evidence of a validated disease mechanism.

Among the upregulated genes, only *CD44* showed significant differential expression and was also present in the core AD interactome. Its increased expression in elderly individuals with AD is consistent with the previously described involvement of *CD44* in inflammatory and tissue-remodeling processes. Previous evidence has also linked increased *CD44* expression to astrocytic and microglial activation and enhanced neuroinflammatory signaling [78].

Gene expression profiles were also compared between young individuals and elderly with AD, and young and elderly without the disease. Genes and pathways shared between both comparisons were removed from the analysis, so that the remaining results represented molecular alterations preferentially observed in the AD aging comparison.

After this filtering, it was observed that the TGF-β1 signaling pathway (FDR = 0.0164) was enriched among the alterations identified in elderly individuals with AD, but was not detected among the alterations observed in the physiological aging comparison. This dysfunction is consistent with evidence associating decreased TGF-β1 signaling with the accumulation of β-amyloid, exacerbated glial activation, and loss of neuronal homeostasis, events that favor the progression of neurodegeneration characteristic of AD [79–81]. In contrast, experimental studies have reported that restoration of TGF-β1 activity can reduce Aβ deposition and chronic inflammation [82, 83]. These findings provide biological context for the pathway enrichment observed in the present analysis, although therapeutic effects of TGF-β1 modulation were not evaluated in the present study.

Also, differentially expressed genes observed only in healthy elderly showed significant enrichment in the HIF-1 signaling pathway (FDR = 0.0436). This result is consistent with evidence linking HIF-1 signaling to cellular responses to oxidative stress and iron homeostasis [84, 85]. However, the present analysis does not establish whether the observed enrichment reflects a protective response or a direct functional role during physiological aging. The HIF-1 pathway acts in regulating the cellular response to oxidative stress and iron homeostasis, processes closely related to neuronal function and synaptic integrity [84, 85]. Controlled activation of HIF-1 stimulates the expression of genes such as *TfR1*, responsible for iron transport, ensuring the balance between uptake and storage of the metal [84, 86].

Accumulating evidence indicates that sustained HIF-1 activation in AD models and AD brain tissue is associated with alterations in iron metabolism, oxidative stress, and related pathological processes [84–87]. These findings provide biological context for the HIF-1 enrichment observed in the present analysis. However, HIF-1 pathway activity and its downstream functional effects were not directly assessed in this study. Experimental evidence has also linked dysregulated iron homeostasis to increased reactive oxygen species (ROS) production, oxidative stress, and processes related to β-amyloid accumulation and tau hyperphosphorylation [86, 87]. Together, these findings provide a potential biological context for the differences observed between physiological aging and AD-related transcriptomic alterations, although the present analysis does not establish a direct functional role for HIF-1 in these processes.

Overall, the comparison between the physiological aging and AD aging groups identified differential enrichment of TGF-β1 and HIF-1-related pathways across the analyzed groups. These findings are consistent with previous evidence linking these pathways to processes involved in neural homeostasis, but do not establish a direct functional role for either pathway in the present dataset. While the TGF-β1 pathway shows negative dysregulation in individuals with AD, the HIF-1 pathway remained relatively preserved in elderly controls, a pattern that may be consistent with previously described responses to age-associated oxidative and inflammatory stress.

### Network Module Prioritization and Functional Enrichment Analyses

After the analyses of the comparisons performed using the transcriptomic datasets, a cluster analysis was conducted using the general AD interactome network (Table S4 in the Supplementary Material). This approach allowed the identification of groups of functionally related genes and the integration of the previously described pathways, offering a systemic view of the most relevant biological processes in the disease pathogenesis.

Clusters 2 and 8 were prioritized for downstream analyses because they were the only network modules containing genes that remained after the filtering procedure designed to identify molecular alterations preferentially detected in the AD aging comparison relative to the physiological aging comparison. Therefore, these clusters represented candidate molecular alterations preferentially observed in the AD aging comparison within the analytical framework adopted in this study. The remaining clusters were not prioritized because they did not contain genes retained after the aging-comparison filtering procedure, and were not considered directly relevant to the objectives of this specific analysis.

Cluster 2 presented 44 interacting genes, of which three were overexpressed in individuals with AD. Functional enrichment analysis of the cluster indicated a predominance of the PI3K-Akt and NF-κB pathways, both associated with inflammatory signaling and cell survival [51, 57, 88, 89]. Among the overexpressed genes, *EGFR* is also involved in signaling processes that can regulate the PI3K/Akt pathway and has been implicated in cellular responses under neurodegenerative conditions [90]. Its identification within the prioritized cluster therefore provides biological context for the observed enrichment of PI3K/Akt-related signaling. However, *EGFR* or PI3K/Akt pathway activation was not directly assessed in the present study.

The NF-κB pathway has been widely implicated in AD pathophysiology and neuroinflammatory processes [91]. Dysregulation of NF-κB signaling has been associated with inflammatory cytokine production, β-amyloid accumulation, tau hyperphosphorylation, and synaptic dysfunction [92]. In the present analysis, the enrichment of NF-κB-related genes provides transcriptomic evidence consistent with the involvement of inflammatory signaling in AD, but does not directly establish NF-κB activation or its downstream functional consequences. Thus, the increased expression of genes associated with this pathway is consistent with the presence of persistent inflammatory processes in the transcriptomic profile observed in AD. Modulation of NF-κB-mediated signaling is therefore considered a potential target for therapeutic investigation to reduce inflammation and neuronal damage characteristic of AD [88].

Cluster 8 presented 55 genes, of which four were overexpressed in individuals with AD, with notable participation in MAPK, PI3K-Akt, and NF-κB signaling pathways, all previously discussed and associated with mechanisms of neuroinflammation, cellular stress response, and neuronal death. The overexpression of genes such as *TLR4*, *TAB2*, *FOS*, and *ICAM1* is consistent with alterations in immune-related and inflammatory signaling processes.

*TLR4* is a well-characterized pattern recognition receptor associated with microglial activation and inflammatory mediator release [93, 94]. TAB2 has also been described as an adaptor involved in NF-κB and MAPK signaling, whereas *ICAM1* participates in leukocyte adhesion and inflammatory signaling [95–97]. These previously described functions provide biological context for the enrichment patterns observed in the present analysis, but were not directly assessed here.

**Fig. 4:**
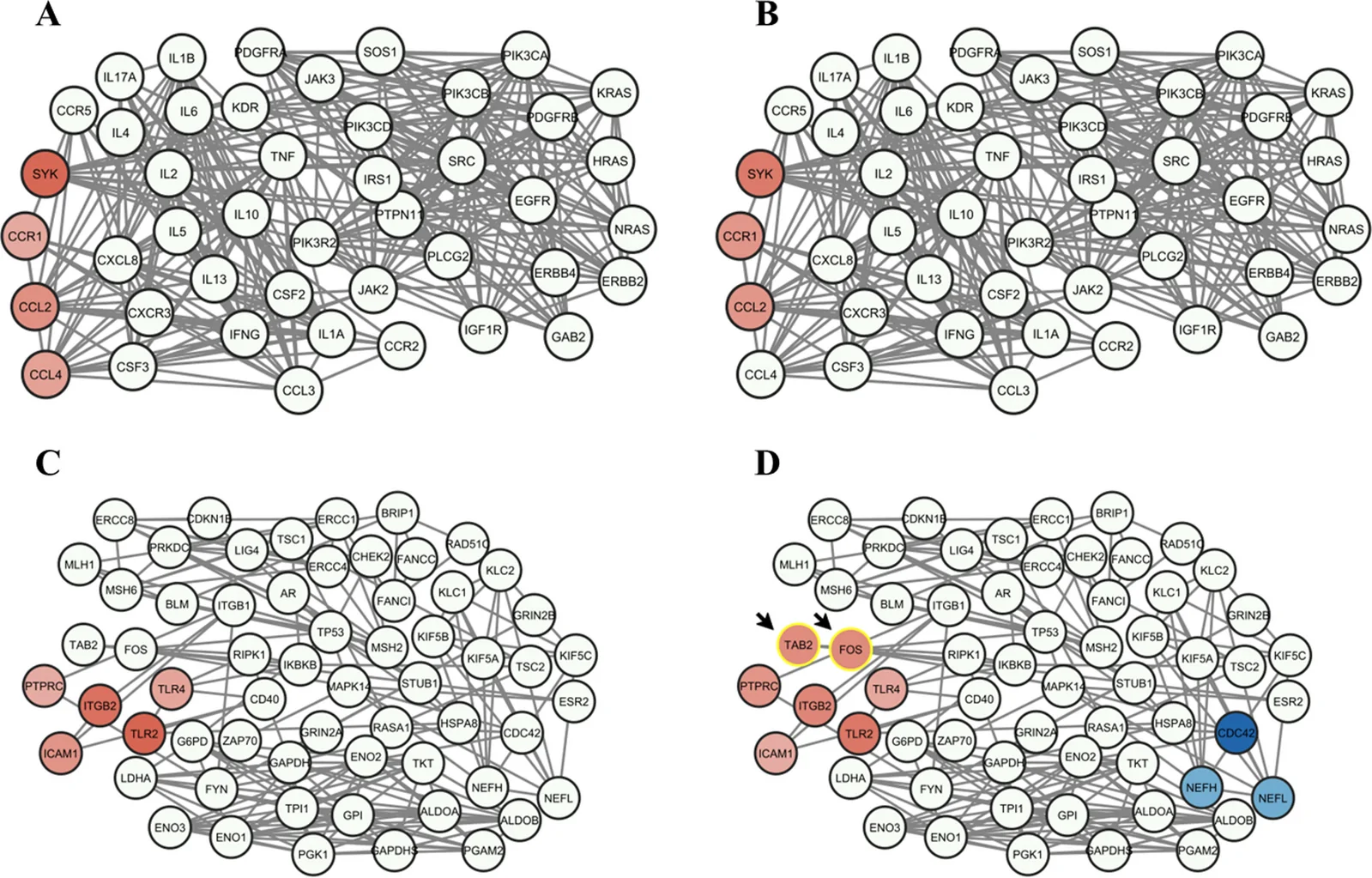
Differential expression of genes belonging to cluster 2 (**A**, **B**) and cluster 8 (**C**, **D**) in the comparisons between elderly without AD and young individuals (**A**, **C**) and elderly with AD and young individuals (**B**, **D**). Nodes represent genes within each cluster and are colored according to the direction and magnitude of differential expression (logFC), with red indicating upregulation and blue indicating downregulation. Only genes meeting the predefined significance criteria (adjusted *p* value/FDR < 0.05) are highlighted. Genes that maintained similar overexpression patterns across both comparisons may represent molecular signatures associated with physiological aging. Arrows indicate *FOS* and *TAB2* (**D**), which were exclusively upregulated in the AD comparison and were therefore prioritized as candidate molecular signatures for further investigation

The comparative analysis of the DEGs between the “elderly without AD vs. young” (Fig. 4A, C) and “elderly with AD vs. young” (Fig. 4B, D) groups revealed a subset of genes consistently upregulated. It was possible to identify upregulated genes present in both comparisons in cluster 2 (Fig. 4A, B), such as *SYK*, *CCL2*, and *CCR1*, and in cluster 8 (Fig. 4C, D), such as *PTPRC*, *ITGB2*, and *TLR2*. These genes possibly reflect normal brain aging processes and are not exclusive to AD.

On the other hand, it was observed that *TLR4* and *ICAM1* do not fit as potential aging markers, as both also showed overexpression in the EOAD vs. CTRL comparison, thus representing candidate molecular signatures preferentially observed in AD-related transcriptomic comparisons. However, *FOS* and *TAB2* (Fig. 4D) maintained increased expression only in the AD condition, suggesting a preferential association with molecular alterations observed in the AD aging comparison within the analytical framework of this study.

Among the genes prioritized through the comparative analysis of physiological aging and the AD aging comparison, *FOS* and *TAB2* emerged as particularly relevant candidate molecular signatures. These genes were detected in the elderly with AD versus young comparison but not in the elderly without AD versus young comparison, suggesting that their transcriptional alterations may be more closely associated with the AD condition than with aging alone. However, because this classification is based on a descriptive comparison of group-specific transcriptomic contrasts rather than a formal age × disease interaction model, these observations should not be interpreted as demonstrating pathological-aging specificity.

Among the genes highlighted in cluster 8, increased expression of *FOS* has been observed in AD models and human tissue, and is associated with neuronal hyperactivity, synaptic loss, and pro-toxic responses to Aβ, indicating a potential role in neurodegenerative progression [98, 99]. On the other hand, *TAB2* is a critical adaptor involved in TAK1–MAPK/NF-κB signaling, and recent experimental studies have implicated aberrant TAK1 activation in AD models [100–103]. These findings provide biological context for the identification of *TAB2* in the present analysis; however, its specific functional contribution to neuroinflammation in human AD remains to be established.

### Integration of Transcriptomic Signatures with Curated Clinical Phenotype Annotations

**Fig. 5:**
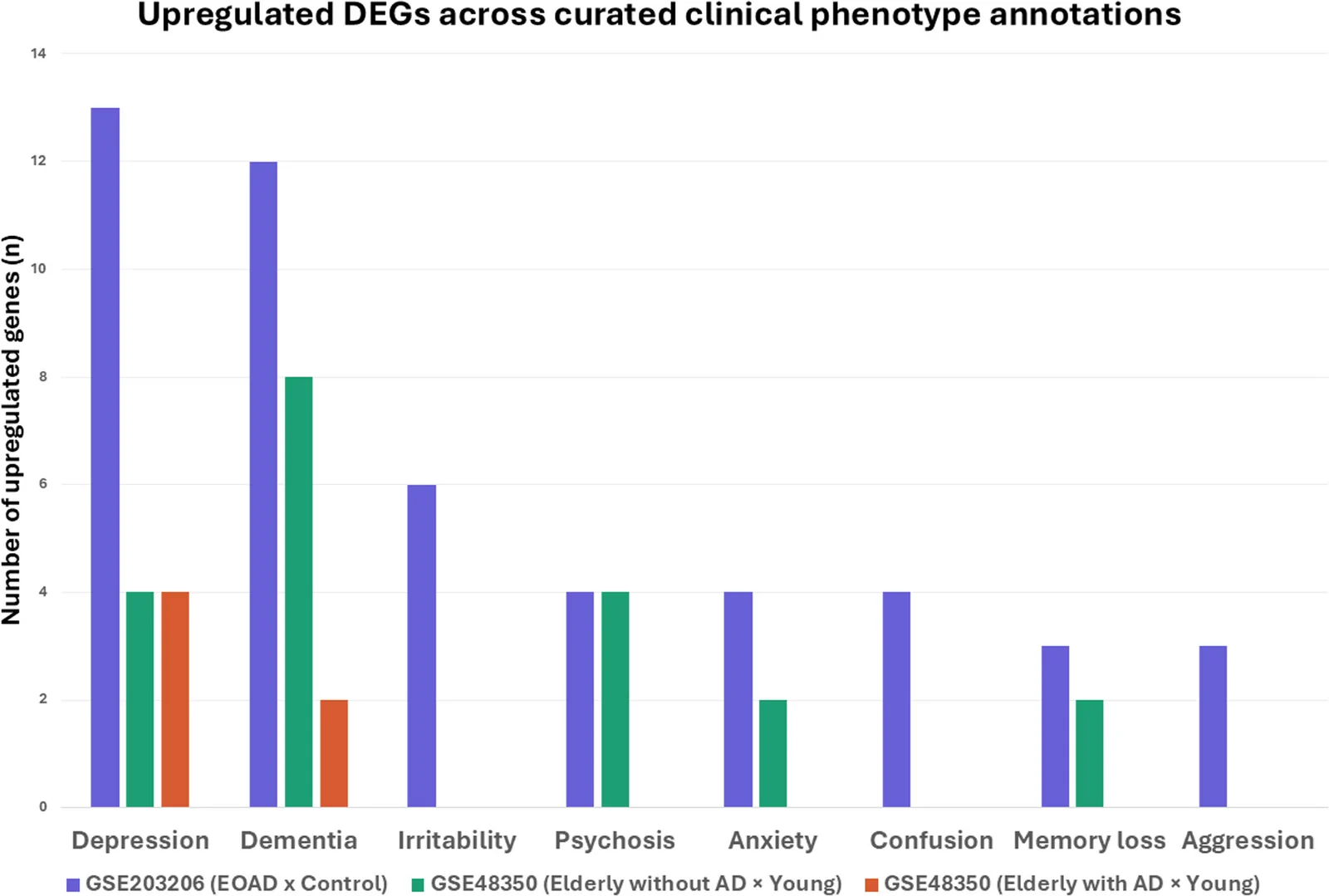
Distribution of upregulated differentially expressed genes across curated clinical phenotype annotations in AD. Bar plots showing the number of upregulated differentially expressed genes (DEGs) identified for each curated clinical phenotype annotation across the analyzed transcriptomic comparisons. Summarized are the overlaps between phenotype-associated gene sets and transcriptomic signatures detected in the EOAD dataset (GSE203206), physiological aging comparison (elderly without AD vs. young individuals), and pathological aging comparison (elderly with AD vs. young individuals) from GSE48350

The final analysis aimed to characterize the overlap between 21 curated clinical phenotype annotations, the AD interactome, and differentially expressed genes identified in the transcriptomic datasets. This approach revealed gene overlaps between the AD interactome, transcriptomic signatures, and several curated clinical phenotype annotations. To formally assess whether phenotype-associated genes were enriched among the DEGs, Fisher’s exact tests and Jaccard index were performed using the 2706-gene AD interactome as the background gene set. While some characteristics, such as depression and dementia, presented larger numbers of genes in common with the interactome (304 and 446, respectively), others, such as sundowning syndrome and eating difficulties, showed a smaller overlap, of only one and eight genes, respectively. For this analysis, we focused on upregulated differentially expressed genes because they represented the main transcriptional signatures associated with the biological conditions of interest in the present study, including EOAD, physiological aging, and pathological aging. For the graphical summary, we displayed the eight curated clinical phenotype annotations with the highest number of overlapping upregulated DEGs across the analyzed transcriptomic comparisons. This selection was made to improve visualization and highlight the phenotype annotations with the strongest overlap patterns (Fig. 5).

Across the 84 phenotype–transcriptomic overlap tests, none remained statistically significant after Benjamini–Hochberg correction (BH-FDR < 0.05). Moreover, we had a range of 0.0032 and 0.0344 Jaccard index between all clinical phenotype annotations. These results indicate that, although phenotype-associated genes showed varying degrees of overlap with the DEG sets across the analyzed transcriptomic contrasts, these overlaps did not show statistically significant enrichment after accounting for the background gene set and multiple testing. Therefore, the observed overlaps should be interpreted as exploratory and hypothesis-generating rather than as statistically supported enrichment between specific phenotype-associated gene sets and transcriptomic alterations.

Complete results for all 21 curated clinical phenotype annotations, including the number of genes overlapping with the AD interactome and the number of overlapping DEGs identified in each transcriptomic comparison, are provided in Table S9 of the Supplementary Material.

Furthermore, the search for differentially expressed genes within these clinical associations, based on the comparisons performed with the datasets, revealed the presence of differentially expressed genes overlapping with several curated clinical phenotype annotations, including memory loss, aggressiveness, seizure, and psychosis, among others. The characteristics personality changes, wandering, stereotyped movement, sundowning syndrome, distrust, euphoria, sleep disorders, and eating difficulties did not present differentially expressed genes. These findings reinforce the molecular complexity underlying AD and suggest that different phenotype annotations showed distinct patterns of gene overlap across the analyzed transcriptomic signatures.

Beyond the quantitative analysis, a qualitative evaluation of the upregulated differentially expressed genes identified in the EOAD versus control comparison revealed several genes that recurred across multiple curated clinical phenotype annotations, including *NOS3*, *HLA-DQB1*, and *ATP1A2*. These recurrent overlaps were considered candidate molecular intersections rather than statistically validated gene–phenotype associations. These recurrent overlaps included *NOS3* (aggression, anxiety, dementia, memory loss, and psychosis), *HLA-DQB1* (anxiety, apathy, dementia, depression, and irritability), and *ATP1A2* (anxiety, confusion, seizures, and depression).

The *NOS3* gene, fundamental for vascularity and endothelial homeostasis [104], overlapped with curated phenotype annotations related to behavioral and cognitive characteristics such as aggression, anxiety, dementia, memory loss, and psychosis. This broad phenotype annotation profile is consistent with previous studies linking dysfunctions in the nitric oxide pathway to neurovascular components of AD, mood disorders, and cognitive impairments [105–108]. Particularly noteworthy was the overlap between *NOS3* and phenotype annotations related to psychosis, a relationship that has been previously explored in the literature, although with conflicting findings. Therefore, the identification of *NOS3* across multiple curated clinical phenotype annotations highlights *NOS3* as a candidate for further investigation in the context of neuropsychiatric phenotype annotations in AD.

The *HLA-DQB1* gene, a central component of the *Major Histocompatibility Complex* class II and therefore essential for antigen presentation and adaptive immune response [109], overlapped with phenotype-associated gene sets related to anxiety, apathy, dementia, depression, and irritability. The identification of *HLA-DQB1* across multiple curated clinical phenotype annotations is consistent with the neuroimmunological hypothesis of AD and with evidence suggesting that HLA variants may influence both disease susceptibility and behavioral phenotypes [110–112].

Finally, the *ATP1A2* gene, crucial for neuronal ionic gradient and cellular excitability [113], displayed overlap across phenotype-associated gene sets representing anxiety, confusion, seizure, and depression. Experimental evidence shows that partial *ATP1A2* deficiency in murine models results in increased anxious behaviors [114], accompanied by neuronal hyperexcitability and alterations in glutamatergic transmission [115]. Previous studies suggest that Na+/K+-ATPase dysfunction may affect neuronal circuits involved in emotional regulation. These observations are compatible with the hypothesis that alterations in ionic homeostasis may contribute to biological processes linked to multiple phenotype annotations.

Collectively, these findings indicate that genes recurring across multiple curated clinical phenotype annotations were functionally related to inflammatory, metabolic, neurovascular, and neuronal excitability-related processes. Within the knowledge-based framework applied in this study, these recurrent genes may represent candidate molecular intersection points across distinct phenotype annotation categories. However, these overlaps do not demonstrate direct molecular mechanisms underlying individual symptoms. Thus, the integration of the AD interactome, differential expression profiles, and curated clinical phenotype annotations provides a hypothesis-generating framework for prioritizing candidate molecular signatures that require clinical and experimental validation.

### Independent Validation of Prioritized Transcriptomic Signatures in an External Dataset

To assess the reproducibility and directional consistency of the gene expression profiles identified in the main analyses, an independent validation was performed using the GSE122063 dataset, consisting of temporal lobe tissue samples from individuals with AD and healthy controls. Twelve candidate genes were selected for focused validation because they had been consistently highlighted across multiple stages of the analytical pipeline, including EOAD differential expression analyses, aging-comparison analyses, signaling pathway enrichment, and endocytosis-related investigations. Importantly, these candidates were defined prior to the focused validation analysis and were not selected based on their statistical significance in the validation dataset. All 12 candidate genes were represented in the independent GSE122063 dataset and were subsequently evaluated for differential expression and directional concordance.

**Fig. 6:**
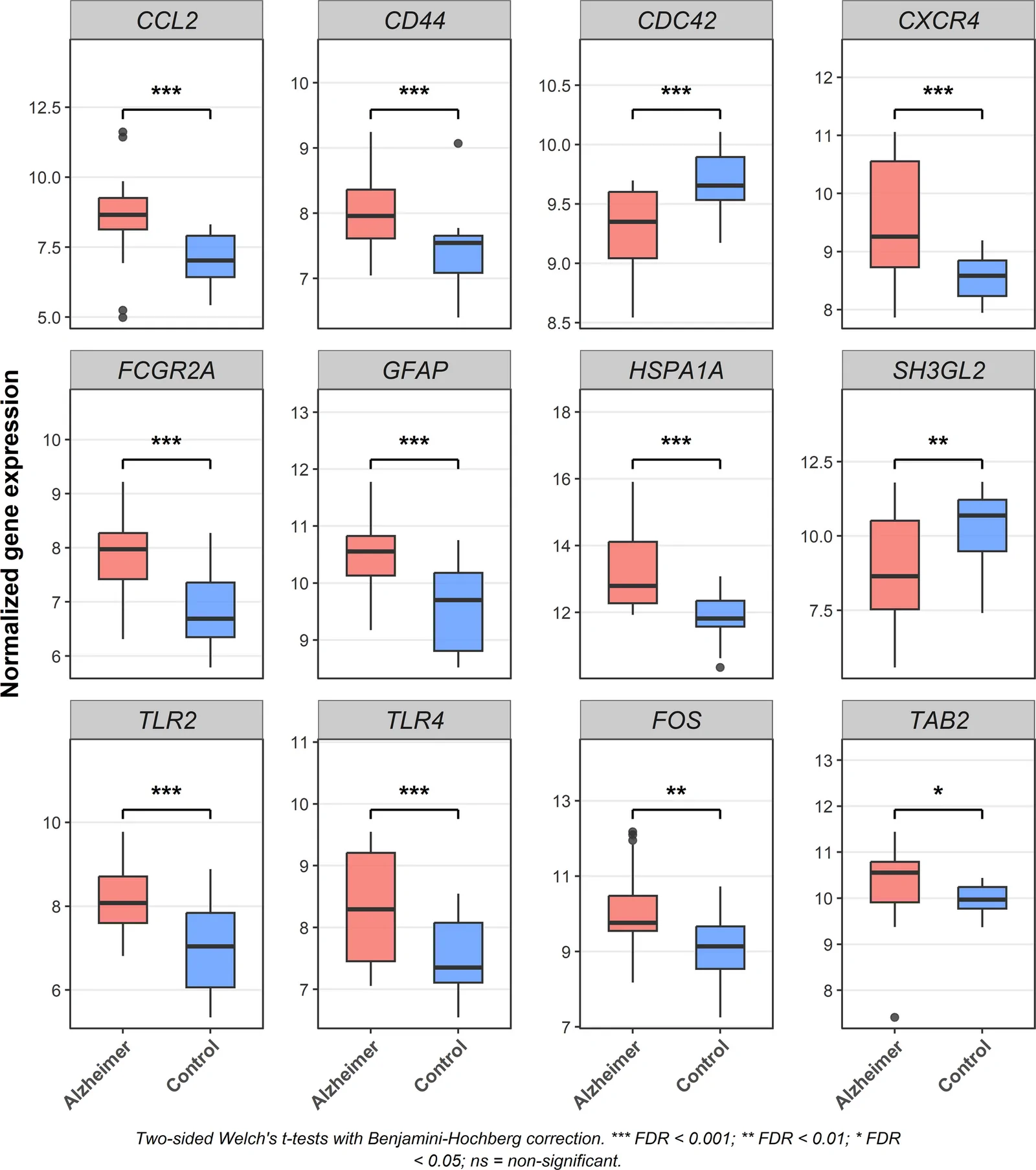
Independent validation of gene expression patterns in the GSE122063 dataset. Boxplots illustrating normalized gene expression values in temporal cortex samples from healthy controls (*N* = 22) and AD patients (*N* = 28). The genes *GFAP*, *CXCR4*, *CCL2*, *HSPA1A*, *FCGR2A*, *TLR2*, *CD44*, *TLR4, FOS*, and *TAB2* showed significantly higher expression levels in the AD group, whereas *CDC42* and *SH3GL2* exhibited lower expression levels. Statistical comparisons between groups were performed using a two-sided Welch’s *t*-test for genes with normally distributed expression values in both groups (Shapiro–Wilk test, *p *> 0.05); otherwise, a two-sided Wilcoxon rank-sum test with continuity correction was applied. *p* values were adjusted for multiple testing using the Benjamini–Hochberg procedure across the 12 tested genes. Levels of statistical significance are indicated as follows: ns, not significant; *p < 0.05; **p < 0.01; ***p < 0.001. Statistical significance indicators correspond to FDR-adjusted *p* values

The expression analysis showed concordant regulation patterns for all 12 previously prioritized candidates, consistent with those observed in the GSE203206 and GSE48350 datasets. Specifically, *GFAP*, *CXCR4*, *CCL2*, *HSPA1A*, *FCGR2A*, *TLR2*, *CD44*, *FOS*, *TAB2*, and *TLR4* showed significant upregulation, whereas *CDC42* and *SH3GL2* showed significant downregulation in the AD group compared with controls in the validation dataset (Fig. 6). Thus, all 12 candidates met the predefined criteria for concordant expression-level validation. These findings support the reproducibility of the expression patterns of genes previously prioritized across the analytical framework. The selected genes represented biological themes including neuroinflammatory signaling, pathological aging, endocytosis-related processes, and knowledge-based overlaps with curated clinical phenotype annotations. Importantly, validation in the external dataset was restricted to differential expression and directional concordance and should not be interpreted as validation of direct gene–phenotype or gene–symptom relationships.

In a complementary and equally crucial manner, the genes *CDC42* and *SH3GL2* exhibited downregulation in the validation dataset, confirming the pattern observed in the comparison between healthy elderly and elderly with AD (Fig. 6). This finding supports the reproducibility of the observed expression patterns of *CDC42* and *SH3GL2* and provides additional evidence that endocytosis-related transcriptional alterations are reproducible across the analyzed datasets. Nevertheless, the proposed involvement of endocytic dysfunction should still be regarded as an exploratory and hypothesis-generating observation requiring further experimental validation. Given that *CDC42* and *SH3GL2* are fundamental components of endocytic pathways, their consistent downregulation may be compatible with previous evidence linking impaired endocytosis to AD pathology, while preserved endocytic activity has been suggested as a neuroprotective feature of healthy aging.

Because the analyses were based on bulk transcriptomic datasets derived from distinct brain regions, the identified differentially expressed genes may reflect both disease-associated transcriptional alterations and changes in cellular composition, particularly involving reactive astrocytes and microglia. Consequently, the observed expression patterns should be interpreted with caution, since bulk transcriptomic data cannot distinguish between cell-intrinsic regulatory changes and shifts in cell-type abundance. Further validation using single-cell or spatial transcriptomic approaches will be necessary to resolve these effects and confirm the cell-type-specific contribution of these genes to AD pathology. This consideration is particularly relevant for genes such as *GFAP*, *CCL2*, *TLR2*, *TLR4*, and *CD44*, whose increased expression may partially reflect astrocytic and microglial activation, a well-established hallmark of AD, rather than exclusively representing disease-specific transcriptional regulation within individual cell types.

## Conclusion

The present study highlights the importance of integrative bioinformatics approaches in the investigation of Alzheimer’s disease (AD), demonstrating how the combined analysis of transcriptomic data and curated clinical phenotype annotations can identify overlapping molecular signatures across disease-related phenotypic annotations. The inclusion of the clinical characteristics, such as depression, dementia, psychosis, and memory loss, allowed the mapping of molecular signatures to curated clinical phenotype annotations relevant to AD, reinforcing the relevance of multidimensional strategies to understand the complexity of the disease.

The integration of gene expression data and protein–protein interaction networks allowed the prioritization of molecular pathways potentially associated with AD, including MAPK, PI3K/Akt, NF-κB, and TGF-β1 signaling pathways. Comparisons between the physiological aging and AD aging groups distinguished shared age-related transcriptional changes from candidate molecular signatures preferentially observed in the AD aging comparison. Within the analyzed comparisons, *FOS* and *TAB2* were upregulated in the elderly with AD versus young comparison but not in the elderly without AD versus young comparison, suggesting that these genes may be more closely associated with the AD condition than with aging alone. In contrast, genes upregulated in both elderly groups may reflect molecular processes associated with brain aging.

Importantly, the systematic integration of molecular signatures with curated clinical phenotype annotations suggested that distinct AD-related phenotypic features may share partially overlapping biological patterns. This knowledge-based framework provides a broader perspective on potential relationships between molecular alterations and disease-associated phenotype annotations while preserving phenotype-specific molecular characteristics.

A major strength of this study is the integration of multiple analytical layers within a unified framework, including disease-associated interactome reconstruction, differential transcriptomic analyses, pathological aging assessment, network-based prioritization, independent expression validation, and curated clinical phenotype annotations. Rather than focusing exclusively on canonical hub genes, isolated transcriptomic findings, or network topology, this integrative strategy enabled the prioritization of candidate molecular signatures across distinct biological and phenotypic dimensions of AD, including candidate molecular signatures preferentially observed in the AD aging comparison (*FOS* and *TAB2*) and candidate endocytosis-associated genes (*CDC42* and *SH3GL2*).

Several limitations of the present study should be acknowledged. First, the transcriptomic analyses were based on publicly available datasets with heterogeneous metadata availability, which prevented the systematic incorporation of covariates such as age, biological sex, disease severity, postmortem interval, and batch effects into unified statistical models. In addition, the analyzed datasets were generated using distinct experimental platforms and derived from different brain regions, including hippocampus, primary visual cortex, and temporal lobe tissue, which differ in cellular composition, baseline transcriptional programs, and susceptibility to AD pathology. Although each dataset was analyzed independently to minimize technical bias, regional and platform-specific effects may still influence the observed molecular signatures. Furthermore, formal sensitivity analyses using alternative threshold values were not performed. Therefore, although the selected cutoffs were based on biological relevance and commonly adopted statistical criteria, some results may vary under different parameter settings. Finally, because the study was based on transcriptomic, network-based, and knowledge-driven computational analyses, the identified associations should be interpreted as hypothesis-generating. Furthermore, the curated clinical phenotype annotations were derived from literature and database resources rather than from patient-level clinical measurements. Consequently, the identified gene–phenotype overlaps may be influenced by annotation density, literature availability, and prior knowledge biases and should not be interpreted as direct clinical associations.

## Supplementary Information

Below is the link to the electronic supplementary material.

ESM 1 (XLSX 1.00 MB)

## Data Availability

Data supporting the findings in this study are included within the Supplementary File. Gene expression data analyzed during the current study are available in the Gene Expression Omnibus (GEO) repository under persistent accession numbers GSE203206, GSE48350 and GSE122063.

## References

[1] Serrano-Pozo A, Frosch MP, Masliah E, Hyman BT (2011) Neuropathological alterations in Alzheimer disease. Cold Spring Harb Perspect Med 1:a006189. 10.1101/CSHPERSPECT.A006189 PMC3234452 22229116

[2] Mayeux R, Stern Y (2012) Epidemiology of Alzheimer disease. Cold Spring Harb Perspect Med 2:a006239. 10.1101/CSHPERSPECT.A006239 PMC3405821 22908189

[3] Jahn H (2013) Memory loss in Alzheimer’s disease. Dialogues Clin Neurosci 15:445–454. 10.31887/DCNS.2013.15.4/HJAHN PMC3898682 24459411

[4] Pless A, Ware D, Saggu S et al (2023) Understanding neuropsychiatric symptoms in Alzheimer’s disease: challenges and advances in diagnosis and treatment. Front Neurosci 17:1263771. 10.3389/FNINS.2023.1263771/FULL PMC10508352 37732300

[5] Raskin J, Cummings J, Hardy J et al (2015) Neurobiology of Alzheimer’s disease: integrated molecular, physiological, anatomical, biomarker, and cognitive dimensions. Curr Alzheimer Res 12:712–722. 10.2174/1567205012666150701103107/CITE/REFWORKS PMC5384474 26412218

[6] Yu R, Topiwala A, Jacoby R, Fazel S (2019) Aggressive behaviors in Alzheimer disease and mild cognitive impairment: systematic review and meta-analysis. Am J Geriatr Psychiatry 27:290–300. 10.1016/J.JAGP.2018.10.008 PMC6399100 30527275

[7] Teixeira AL, Rocha NP, Gatchel J (2023) Behavioral or neuropsychiatric symptoms of Alzheimer’s disease: from psychopathology to pharmacological management. Arq Neuropsiquiatr 81:1152–1162. 10.1055/S-0043-1777774/ID/JR230228-36/BIB PMC10756775 38157881

[8] Koenig AM, Arnold SE, Streim JE (2016) Agitation and irritability in Alzheimer’s disease: evidenced-based treatments and the black-box warning. Curr Psychiatry Rep 18:1–10. 10.1007/S11920-015-0640-7/METRICS PMC6483820 26695173

[9] Van DD, Vermeiren Y, Dekker AD et al (2016) Neuropsychiatric disturbances in Alzheimer’s disease: what have we learned from neuropathological studies? Curr Alzheimer Res 13:1145–1164. 10.2174/1567205013666160502123607/CITE/REFWORKS PMC5070416 27137218

[10] Sultzer DL, Leskin LP, Melrose RJ et al (2014) Neurobiology of delusions, memory, and insight in Alzheimer disease. Am J Geriatr Psychiatry 22:1346–1355. 10.1016/J.JAGP.2013.06.005 PMC4254898 24021220

[11] Friedman D, Honig LS, Scarmeas N (2012) Seizures and epilepsy in Alzheimer’s disease. CNS Neurosci Ther 18:285–294. 10.1111/J.1755-5949.2011.00251.X PMC3630499 22070283

[12] Goodwin GJ, Moeller S, Nguyen A et al (2023) Network analysis of neuropsychiatric symptoms in Alzheimer’s disease. Alzheimer’s Res Ther 15(1):135. 10.1186/S13195-023-01279-6 PMC10416506 37568209

[13] Canevelli M, Valletta M, Trebbastoni A et al (2016) Sundowning in dementia: Clinical relevance, pathophysiological determinants, and therapeutic approaches. Front Med (Lausanne) 3:237904. 10.3389/FMED.2016.00073/BIBTEX PMC5187352 28083535

[14] Polin C, Gellé T, Auditeau E et al (2023) Repetitive behaviors in Alzheimer’s disease: a systematic review and meta-analysis. J Alzheimer’s Dis 96:483–497. 10.3233/JAD-230380;PAGE:STRING:ARTICLE/CHAPTER 37781801

[15] Lopes KF, Bahia VS, Natividade JC et al (2022) Changes in personality traits in patients with Alzheimer’s disease. Dement Neuropsychol 16:187–193. 10.1590/1980-5764-DN-2021-0029 PMC9173796 35720659

[16] Kai K, Hashimoto M, Amano K et al (2015) Relationship between eating disturbance and dementia severity in patients with Alzheimer’s disease. PLoS ONE 10:e0133666. 10.1371/JOURNAL.PONE.0133666 PMC4534398 26266531

[17] Thomas T, Ritter A (2022) Wandering & sundowning in dementia. Practical Neurology. https://practicalneurology.com/diseases-diagnoses/alzheimer-disease-dementias/wandering-sundowning-in-dementia/31912/. Accessed 3 Mar 2026

[18] Kakkar A, Singh H, Anand A et al (2025) A comprehensive review of genetic risk factors for Alzheimer’s disease development. Curr Gene Ther 25. 10.2174/0115665232397101250916050247 40990281

[19] Mulugeta A, Navale SS, Lumsden AL et al (2022) Healthy lifestyle, genetic risk and brain health: a gene-environment interaction study in the UK Biobank. Nutrients 14:3907. 10.3390/NU14193907/S1 PMC9570683 36235559

[20] Hasin Y, Seldin M, Lusis A (2017) Multi-omics approaches to disease. Genome Biol 18(1):18–15. 10.1186/S13059-017-1215-1 PMC5418815 28476144

[21] Zhang Q, Ma C, Gearing M et al (2018) Integrated proteomics and network analysis identifies protein hubs and network alterations in Alzheimer’s disease. Acta Neuropathol Commun 6(1):6–19. 10.1186/S40478-018-0524-2 PMC5831854 29490708

[22] de Carvalho LM, Carvalho VMA, Camargo AP, Papes F (2025) Gene network analysis identifies dysregulated pathways in an autism spectrum disorder caused by mutations in transcription factor 4. Sci Rep 15(1):4993. 10.1038/s41598-025-89334-0 PMC11811132 39929970

[23] Sanches PHG, de Melo NC, Porcari AM, de Carvalho LM (2024) Integrating molecular perspectives: strategies for comprehensive multi-omics integrative data analysis and machine learning applications in transcriptomics, proteomics, and metabolomics. Biology (Basel) 13:848. 10.3390/biology13110848 PMC11592251 39596803

[24] Hampel H, Nisticò R, Seyfried NT et al (2021) Omics sciences for systems biology in Alzheimer’s disease: state-of-the-art of the evidence. Ageing Res Rev 69:101346. 10.1016/J.ARR.2021.101346 33915266

[25] Mangleburg CG, Wu T, Yalamanchili HK et al (2020) Integrated analysis of the aging brain transcriptome and proteome in tauopathy. Mol Neurodegener 15(1):56. 10.1186/S13024-020-00405-4 PMC7526226 32993812

[26] Grewal A, Sheokand D, Chauhan R et al (2024) Insights into amyloid precursor protein target through PPI network analysis. Bioinformation 20:140. 10.6026/973206300200140 PMC10941771 38497073

[27] Karbalaei R, Allahyari M, Rezaei-Tavirani M et al (2018) Protein-protein interaction analysis of Alzheimer`s disease and NAFLD based on systems biology methods unhide common ancestor pathways. Gastroenterol Hepatol Bed Bench 11:27. 10.22037/ghfbb.v0i0.1327 PMC5849115 29564062

[28] Kumar V, Roy K (2024) Protein-protein interaction network analysis for the identification of novel multi-target inhibitors and target miRNAs against Alzheimer’s disease. Adv Protein Chem Struct Biol 139:405–467. 10.1016/BS.APCSB.2023.11.005 38448142

[29] Patel H, Dobson RJB, Newhouse SJ (2019) A meta-analysis of Alzheimer’s disease brain transcriptomic data. J Alzheimers Dis 68:1635–1656. 10.3233/JAD-181085 PMC6484273 30909231

[30] Morabito S, Miyoshi E, Michael N, Swarup V (2020) Integrative genomics approach identifies conserved transcriptomic networks in Alzheimer’s disease. Hum Mol Genet 29:2899–2919. 10.1093/HMG/DDAA182 PMC7566321 32803238

[31] Neff RA, Wang M, Vatansever S et al (2021) Molecular subtyping of Alzheimer’s disease using RNA sequencing data reveals novel mechanisms and targets. Sci Adv. 10.1126/SCIADV.ABB5398 PMC7787497 33523961

[32] Fisher DW, Dunn JT, Keszycki R et al (2024) Unique transcriptional signatures correlate with behavioral and psychological symptom domains in Alzheimer’s disease. Transl Psychiatry 14(1):178. 10.1038/s41398-024-02878-z PMC10995139 38575567

[33] Ceylan B, Düz E, Çakir T (2024) Personalized protein-protein interaction networks towards unraveling the molecular mechanisms of Alzheimer’s disease. Mol Neurobiol 61:2120–2135. 10.1007/S12035-023-03690-4 37855983

[34] Kodam P, Sai Swaroop R, Pradhan SS et al (2023) Integrated multi-omics analysis of Alzheimer’s disease shows molecular signatures associated with disease progression and potential therapeutic targets. Sci Rep 13(1):3695. 10.1038/s41598-023-30892-6 PMC9986671 36879094

[35] Zhang Z, Liu X, Zhang S et al (2024) A review and analysis of key biomarkers in Alzheimer’s disease. Front Neurosci 18:1358998. 10.3389/FNINS.2024.1358998/FULL PMC10912539 38445255

[36] Szklarczyk D, Franceschini A, Wyder S et al (2015) STRING v10: protein–protein interaction networks, integrated over the tree of life. Nucleic Acids Res 43:D447–D452. 10.1093/NAR/GKU1003 PMC4383874 25352553

[37] Shannon P, Markiel A, Ozier O et al (2003) Cytoscape: a software environment for integrated models of biomolecular interaction networks. Genome Res 13:2498. 10.1101/GR.1239303 PMC403769 14597658

[38] Harris MA, Clark J, Ireland A et al (2004) The Gene Oncology (GO) database and informatics resource. Nucleic Acids Res 32. 10.1093/nar/gkh036 PMC308770 14681407

[39] Kanehisa M, Goto S (2000) KEGG: Kyoto Encyclopedia of Genes and Genomes. Nucleic Acids Res 28:27–30 10.1093/nar/28.1.27 PMC102409 10592173

[40] Sherman BT, Hao M, Qiu J et al (2022) DAVID: a web server for functional enrichment analysis and functional annotation of gene lists (2021 update). Nucleic Acids Res 50:W216–W221. 10.1093/nar/gkac194 PMC9252805 35325185

[41] Reichenbach N, Delekate A, Plescher M et al (2019) Inhibition of Stat3‐mediated astrogliosis ameliorates pathology in an Alzheimer’s disease model. EMBO Mol Med 11. 10.15252/EMMM.201809665;PAGEGROUP:STRING:PUBLICATION PMC6365929 30617153

[42] Cheng X, Shen Y, Li R (2014) Targeting TNF: a therapeutic strategy for Alzheimer’s disease. Drug Discov Today 19:1822–1827. 10.1016/J.DRUDIS.2014.06.029 24998784

[43] Jin D, Zhang M, Shi L, Liu H (2024) Investigating the impact of IL-6 and CXCL8 on neurodegeneration and cognitive decline in Alzheimer disease. Int J Neuropsychopharmacol 28:38. 10.1093/IJNP/PYAE038 PMC11781222 39223908

[44] Mastroeni D, Chan CK, Morshed N et al (2024) Upregulation of the proto-oncogene Src kinase in Alzheimer’s disease: from molecular interactions to therapeutic potential. bioRxiv 2024.02.07.579336. 10.1101/2024.02.07.579336

[45] Rosmanah L, Saepuloh U, Mariya SS et al (2024) Expression of APP, CDK5, and AKT1 gene related to Alzheimer disease in brain of long-tailed macaques. Hayati J Biosci 31:145–152. 10.4308/HJB.31.1.145-152

[46] Pang S, Li S, Cheng H et al (2023) Discovery of an evodiamine derivative for PI3K/AKT/GSK3β pathway activation and AD pathology improvement in mouse models. Front Mol Neurosci 15:1025066. 10.3389/FNMOL.2022.1025066/BIBTEX PMC9868638 36698780

[47] Zhuang W, Ye T, Wang W et al (2023) CTNNB1 in neurodevelopmental disorders. Front Psychiatry 14:1143328. 10.3389/FPSYT.2023.1143328/BIBTEX PMC10061110 37009120

[48] Wolfrum P, Fietz A, Schnichels S, Hurst J (2022) The function of p53 and its role in Alzheimer’s and Parkinson’s disease compared to age-related macular degeneration. Front Neurosci 16:1029473. 10.3389/FNINS.2022.1029473/BIBTEX PMC9811148 36620455

[49] Astillero-Lopez V, Villar-Conde S, Gonzalez-Rodriguez M et al (2024) Proteomic analysis identifies HSP90AA1, PTK2B, and ANXA2 in the human entorhinal cortex in Alzheimer’s disease: potential role in synaptic homeostasis and Aβ pathology through microglial and astroglial cells. Brain Pathol 34:e13235. 10.1111/BPA.13235;PAGEGROUP:STRING:PUBLICATION PMC11189773 38247340

[50] Choi HJ, Jeong YJ, Kim J, Hoe HS (2023) EGFR is a potential dual molecular target for cancer and Alzheimer’s disease. Front Pharmacol 14:1238639. 10.3389/FPHAR.2023.1238639/FULL PMC10433764 37601068

[51] Razani E, Pourbagheri-Sigaroodi A, Safaroghli-Azar A et al (2021) The PI3K/Akt signaling axis in Alzheimer’s disease: a valuable target to stimulate or suppress? Cell Stress Chaperones 26:871–887. 10.1007/S12192-021-01231-3 PMC8578535 34386944

[52] Lin H, Dixon SG, Hu W et al (2022) P38 MAPK is a major regulator of amyloid beta-induced IL-6 expression in human microglia. Mol Neurobiol 59:5284–5298. 10.1007/S12035-022-02909-0 PMC9398979 35697992

[53] Chen MJ, Ramesha S, Weinstock LD et al (2021) Extracellular signal-regulated kinase regulates microglial immune responses in Alzheimer’s disease. J Neurosci Res 99:1704–1721. 10.1002/JNR.24829 PMC8919593 33729626

[54] Chen Y, Yu Y (2023) Tau and neuroinflammation in Alzheimer’s disease: interplay mechanisms and clinical translation. J Neuroinflammation 20:1–21. 10.1186/S12974-023-02853-3/TABLES/1 PMC10349496 37452321

[55] Li X, Teng T, Yan W et al (2023) AKT and MAPK signaling pathways in hippocampus reveals the pathogenesis of depression in four stress-induced models. Transl Psychiatry 13. 10.1038/S41398-023-02486-3 PMC10261007 37308476

[56] Amini J, Beyer C, Zendedel A, Sanadgol N (2023) MAPK is a mutual pathway targeted by anxiety-related miRNAs, and E2F5 is a putative target for anxiolytic miRNAs. Biomolecules 13:544. 10.3390/BIOM13030544 PMC10046777 36979479

[57] Pan J, Yao Q, Wang Y et al (2024) The role of PI3K signaling pathway in Alzheimer’s disease. Front Aging Neurosci 16:1459025. 10.3389/FNAGI.2024.1459025/FULL PMC11466886 39399315

[58] Lauretti E, Dincer O, Praticò D (2020) Glycogen synthase kinase-3 signaling in Alzheimer’s disease. Biochim Biophys Acta (BBA) Mol Cell Res 1867:118664. 10.1016/J.BBAMCR.2020.118664 PMC7047718 32006534

[59] Mahoney ER, Dumitrescu L, Moore AM et al (2021) Brain expression of the vascular endothelial growth factor gene family in cognitive aging and Alzheimer’s disease. Mol Psychiatry 26:888–896. 10.1038/S41380-019-0458-5;TECHMETA PMC6980445 31332262

[60] Guo N, Wang X, Xu M et al (2024) PI3K/AKT signaling pathway: molecular mechanisms and therapeutic potential in depression. Pharmacol Res 206:107300. 10.1016/J.PHRS.2024.107300 38992850

[61] Wang L, Lu D, Wang X et al (2024) The effects of nitric oxide in Alzheimer’s disease. Med Gas Res 14:186–191. 10.4103/2045-9912.385939 PMC11257180 39073326

[62] Iova OM, Marin GE, Lazar I et al (2023) Nitric oxide/nitric oxide synthase system in the pathogenesis of neurodegenerative disorders—an overview. Antioxidants (Basel) 12:753. 10.3390/ANTIOX12030753 PMC10045816 36979000

[63] Wright JW, Harding JW (2015) The brain hepatocyte growth factor/c-Met receptor system: a new target for the treatment of Alzheimer’s disease. J Alzheimers Dis 45:985–1000. 10.3233/JAD-142814 25649658

[64] Mi X, Jiao W, Yang Y et al (2022) HGF secreted by mesenchymal stromal cells promotes primordial follicle activation by increasing the activity of the PI3K-AKT signaling pathway. Stem Cell Rev Rep 18:1834–1850. 10.1007/S12015-022-10335-X/FIGURES/8 PMC9209380 35089464

[65] Wei J, Ma X, Nehme A et al (2022) Reduced HGF/MET signaling may contribute to the synaptic pathology in an Alzheimer’s disease mouse model. Front Aging Neurosci 14:954266. 10.3389/FNAGI.2022.954266/BIBTEX PMC9314739 35903536

[66] Regier NG, Gitlin LN (2018) Dementia-related restlessness: relationship to characteristics of persons with dementia and family caregivers. Int J Geriatr Psychiatry 33:185–192. 10.1002/GPS.4705;JOURNAL:JOURNAL:10991166;WGROUP:STRING:PUBLICATION PMC6964249 28332736

[67] Lei J, Li J, Wu W et al (2025) Neuroprotective effects of Yangming-Kaixin-Yizhi formula in Alzheimer’s disease: dual regulation of PI3K/Akt and p38 MAPK signaling via network pharmacology and experimental approaches. Metab Brain Dis 40(7):1–16. 10.1007/S11011-025-01688-6 40879814

[68] Garrett WS, Chen LM, Kroschewski R et al (2000) Developmental control of endocytosis in dendritic cells by Cdc42. Cell 102:325–334. 10.1016/S0092-8674(00)00038-6 10975523

[69] Wigge P, McMahon HT (1998) The amphiphysin family of proteins and their role in endocytosis at the synapse. Trends Neurosci 21:339–344. 10.1016/S0166-2236(98)01264-8 9720601

[70] Majumdar S, Gong EM, Vizio DD et al (2013) Loss of Sh3gl2/endophilin a1 is a common event in urothelial carcinoma that promotes malignant behavior. Neoplasia N Y 15:749–IN16. 10.1593/NEO.121956 PMC3689238 23814487

[71] Zadka Ł, Sochocka M, Hachiya N et al (2024) Endocytosis and Alzheimer’s disease. Geroscience 46:71–85. 10.1007/S11357-023-00923-1/FIGURES/4 PMC10828383 37646904

[72] Schmid SL (2017) Reciprocal regulation of signaling and endocytosis: implications for the evolving cancer cell. J Cell Biol 216:2623–2632. 10.1083/JCB.201705017 PMC5584184 28674108

[73] El-Sayed A, Harashima H (2013) Endocytosis of gene delivery vectors: from clathrin-dependent to lipid raft-mediated endocytosis. Mol Ther 21:1118–1130. 10.1038/MT.2013.54 PMC3677298 23587924

[74] Shafaq-Zadah M, Dransart E, Johannes L (2020) Clathrin-independent endocytosis, retrograde trafficking, and cell polarity. Curr Opin Cell Biol 65:112–121. 10.1016/J.CEB.2020.05.009 PMC7588825 32688213

[75] Kwart D, Gregg A, Scheckel C et al (2019) A large panel of isogenic APP and PSEN1 mutant human iPSC neurons reveals shared endosomal abnormalities mediated by APP β-CTFs, not Aβ. Neuron 104:256–270.e5. 10.1016/j.neuron.2019.07.010 31416668

[76] Zhao Z, Sagare AP, Ma Q et al (2015) Central role for PICALM in amyloid-β blood-brain barrier transcytosis and clearance. Nat Neurosci 18:978–987. 10.1038/NN.4025;TECHMETA PMC4482781 26005850

[77] Ando K, Brion JP, Stygelbout V et al (2013) Clathrin adaptor CALM/PICALM is associated with neurofibrillary tangles and is cleaved in Alzheimer’s brains. Acta Neuropathol 125:861–878. 10.1007/S00401-013-1111-Z/METRICS 23589030

[78] Zhang M, Lin Y, Wei H et al (2025) The membrane receptor CD44: roles in neurodegenerative diseases. Expert Opin Ther Targets 1–10. 10.1080/14728222.2025.2563243 40958089

[79] Tesseur I, Zou K, Esposito L et al (2006) Deficiency in neuronal TGF-β signaling promotes neurodegeneration and Alzheimer’s pathology. J Clin Invest 116:3060–3069. 10.1172/JCI27341 PMC1626127 17080199

[80] Kapoor M, Chinnathambi S (2023) TGF-β1 signalling in Alzheimer’s pathology and cytoskeletal reorganization: a specialized Tau perspective. J Neuroinflammation 20:1–20. 10.1186/S12974-023-02751-8/FIGURES/6 PMC10012507 36915196

[81] Vidovic N, Spittau B (2024) Microglial transforming growth factor-β signaling in Alzheimer’s disease. Int J Mol Sci 25:3090. 10.3390/IJMS25063090 PMC10970595 38542077

[82] Hu Y, Chen W, Wu L et al (2019) TGF-β1 restores hippocampal synaptic plasticity and memory in Alzheimer model via the PI3K/Akt/Wnt/β-catenin signaling pathway. J Mol Neurosci 67:142–149. 10.1007/S12031-018-1219-7/METRICS 30539409

[83] Chen JH, Ke KF, Lu JH et al (2015) Protection of TGF-β1 against neuroinflammation and neurodegeneration in Aβ1–42-induced Alzheimer’s disease model rats. PLoS ONE 10:e0116549. 10.1371/JOURNAL.PONE.0116549 PMC4319949 25658940

[84] Petralla S, Saveleva L, Kanninen KM et al (2024) Increased expression of transferrin receptor 1 in the brain cortex of 5xFAD mouse model of Alzheimer’s disease is associated with activation of HIF-1 signaling pathway. Mol Neurobiol 61:6383–6394. 10.1007/S12035-024-03990-3/FIGURES/4 PMC11339108 38296900

[85] Lin T-K;, Huang C-R;, Lin K-J; et al (2024) Potential roles of hypoxia-inducible factor-1 in Alzheimer’s disease: beneficial or detrimental? Antioxidants (Basel) 13:1378. 10.3390/ANTIOX13111378 PMC11591038 39594520

[86] Mitroshina EV, Vedunova MV (2024) The role of oxygen homeostasis and the HIF-1 factor in the development of neurodegeneration. Int J Mol Sci 25:4581. 10.3390/IJMS25094581 PMC11083463 38731800

[87] Lei L, Feng J, Wu G et al (2022) HIF-1α causes LCMT1/PP2A deficiency and mediates tau hyperphosphorylation and cognitive dysfunction during chronic hypoxia. Int J Mol Sci 23:16140. 10.3390/IJMS232416140/S1 PMC9783654 36555780

[88] Sivamaruthi BS, Raghani N, Chorawala M et al (2023) NF-κB pathway and its inhibitors: a promising frontier in the management of Alzheimer’s disease. Biomedicines Basel 11:2587. 10.3390/BIOMEDICINES11092587 PMC10526355 37761028

[89] Limantoro J, de Liyis BG, Sutedja JC (2023) Akt signaling pathway: a potential therapy for Alzheimer’s disease through glycogen synthase kinase 3 beta inhibition. Egypt J Neurol Psychiatry Neurosurg 59:1–14. 10.1186/S41983-023-00751-2/FIGURES/2

[90] Cheng KC (2019) P3–194: the distinct roles of EGFR downstream signaling in Aβ-induced pathologies. Alzheimers Dement 15:P1004–P1004. 10.1016/J.JALZ.2019.06.3223

[91] Sun E, Motolani A, Campos L, Lu T (2022) The pivotal role of NF-kB in the pathogenesis and therapeutics of Alzheimer’s disease. Int J Mol Sci 23:8972. 10.3390/IJMS23168972 PMC9408758 36012242

[92] Hwang CJ, Choi D-Y, Park MH, Hong JT (2017) NF-κB as a key mediator of brain inflammation in Alzheimer’s disease. CNS Neurol Disord Drug Targets 18:3–10. 10.2174/1871527316666170807130011/CITE/REFWORKS 28782486

[93] Song M, Jin JJ, Lim JE et al (2011) TLR4 mutation reduces microglial activation, increases Aβ deposits and exacerbates cognitive deficits in a mouse model of Alzheimer’s disease. J Neuroinflammation 8(1):92. 10.1186/1742-2094-8-92 PMC3169468 21827663

[94] Yang J, Wise L, Fukuchi KI (2020) TLR4 cross-talk with NLRP3 inflammasome and complement signaling pathways in Alzheimer’s disease. Front Immunol 11:526840. 10.3389/FIMMU.2020.00724/FULL PMC7190872 32391019

[95] Takaesu G, Kishida S, Hiyama A et al (2000) TAB2, a novel adaptor protein, mediates activation of TAK1 MAPKKK by linking TAK1 to TRAF6 in the IL-1 signal transduction pathway. Mol Cell 5:649–658. 10.1016/S1097-2765(00)80244-0 10882101

[96] Otgongerel D, Lee HJ, Jo SA (2023) Induction of ICAM1 in brain vessels is implicated in an early AD pathogenesis by modulating neprilysin. Neuromolecular Med 25:193–204. 10.1007/S12017-022-08726-X/METRICS 35948857

[97] Zhang Q, Yang G, Luo Y et al (2024) Neuroinflammation in Alzheimer’s disease: insights from peripheral immune cells. Immunity and Ageing 21:1–25. 10.1186/S12979-024-00445-0/TABLES/3 PMC11177389 38877498

[98] L’Esperance OJ, McGhee J, Davidson G et al (2024) Functional connectivity favors aberrant visual network c-Fos expression accompanied by cortical synapse loss in a mouse model of Alzheimer’s disease. J Alzheimers Dis 101:111–131. 10.3233/JAD-240776 PMC11810533 39121131

[99] Corbett BF, You JC, Zhang X et al (2017) ΔFosB regulates gene expression and cognitive dysfunction in a mouse model of Alzheimer’s disease. Cell Rep 20:344. 10.1016/J.CELREP.2017.06.040 PMC5785235 28700937

[100] Wang C, Deng L, Hong M et al (2001) TAK1 is a ubiquitin-dependent kinase of MKK and IKK. Nature 412(6844):346–351. 10.1038/35085597 11460167

[101] Orelio C, Dzierzak E (2007) Expression analysis of the TAB2 protein in adult mouse tissues. Inflamm Res 56:98–104. 10.1007/S00011-006-6058-2 PMC2770139 17406806

[102] Sai K, Nakanishi A, Scofield KM et al (2023) Aberrantly activated TAK1 links neuroinflammation and neuronal loss in Alzheimer’s disease mouse models. J Cell Sci 136:jcs260102. 10.1242/JCS.260102 PMC10112982 36912451

[103] Zhang W, Xiao D, Mao Q, Xia H (2023) Role of neuroinflammation in neurodegeneration development. Signal Transduct Target Ther 8:1–32. 10.1038/S41392-023-01486-5;SUBJMETA PMC10336149 37433768

[104] Förstermann U, Sessa WC (2012) Nitric oxide synthases: regulation and function. Eur Heart J 33:829–837. 10.1093/EURHEARTJ/EHR304 PMC3345541 21890489

[105] Gutiérrez HC, O’Leary A, Freudenberg F et al (2020) Nitric oxide interacts with monoamine oxidase to modulate aggression and anxiety-like behaviour. Eur Neuropsychopharmacol 30:30–43. 10.1016/J.EURONEURO.2017.09.004 28951000

[106] Liu PL, Song AR, Dong CD et al (2021) Network pharmacology study on the mechanism of the herb pair of prepared Rehmannia root-Chinese arborvitae kernel for anxiety disorders. Ann Palliat Med 10:3313327. 10.21037/APM-21-531 33849116

[107] Manso-Calderón R, Cacabelos-Pérez P, Sevillano-García MD et al (2023) Analysis of endothelial gene polymorphisms in Spanish patients with vascular dementia and Alzheimer’s disease. Sci Rep 13(1):13441. 10.1038/s41598-023-39576-7 PMC10439194 37596325

[108] Taylor G (2024) The interaction between nitric oxide synthase related short and long non-coding RNAs in the context of memory formation, aging and neurogenesis. Doctoral dissertation, University of Sussex. https://hdl.handle.net/10779/uos.25991041.v1. Accessed 3 Mar 2026

[109] National Center for Biotechnology Information (2024) HLA-DQB1 major histocompatibility complex, class II, DQ beta 1 [Homo sapiens (human)]. NCBI Gene. https://www.ncbi.nlm.nih.gov/gene/3119. Accessed 3 Mar 2026

[110] Angelopoulou E, Koros C, Hatzimanolis A et al (2024) Exploring the genetic landscape of mild behavioral impairment as an early marker of cognitive decline: an updated review focusing on Alzheimer’s disease. Int J Mol Sci 25:2645. 10.3390/IJMS25052645 PMC10931648 38473892

[111] Cătană CS, Marta MM, Văleanu M et al (2024) Human leukocyte antigen and microRNAs as key orchestrators of mild cognitive impairment and Alzheimer’s disease: a systematic review. Int J Mol Sci 25:8544. 10.3390/IJMS25158544 PMC11312697 39126112

[112] Shigemizu D, Fukunaga K, Yamakawa A et al (2024) The HLA-DRB1*09:01-DQB1*03:03 haplotype is associated with the risk for late-onset Alzheimer’s disease in APOE {{ }} 4–negative Japanese adults. npj Aging 10(1):3. 10.1038/s41514-023-00131-3 PMC10761915 38167405

[113] National Center for Biotechnology Information (2024) ATP1A2 ATPase Na+/K+ transporting subunit alpha 2 [Homo sapiens (human)]. NCBI Gene. https://www.ncbi.nlm.nih.gov/gene/477. Accessed 3 Mar 2026

[114] Gritz SM, Radcliffe RA (2013) Genetic effects of ATP1A2 in familial hemiplegic migraine type II and animal models. Hum Genomics 7:8. 10.1186/1479-7364-7-8 PMC3639839 23561701

[115] Sugimoto H, Sato M, Nakai J, Kawakami K (2020) Astrocytes in Atp1a2-deficient heterozygous mice exhibit hyperactivity after induction of cortical spreading depression. FEBS Open Bio 10:1031–1043. 10.1002/2211-5463.12848 PMC7262908 32237043

